# Effect of educational intervention on safe traffic behaviors of high school male students in Iran, using the theory of planned behavior: a quasi-experimental study

**DOI:** 10.1186/s12889-021-11943-x

**Published:** 2021-10-18

**Authors:** Vahid Ranaei, Laleh Hassani, Alireza Shahab Jahanlou, Ghodratollah Roshanaei, Forouzan Rezapur-Shahkolai

**Affiliations:** 1 Social Determinants in Health Promotion Research Center, Hormozgan University of Medical Sciences, Bandar Abbas, Iran; 2 School of Health, Social Determinants in Health Promotion Research Center, Hormozgan University of Medical Sciences, Bandar Abbas, Iran; 3 Cardiovascular Research Center, Hormozgan University of Medical Sciences, Bandar Abbas, Iran; 4 Department of Biostatistics, School of Public Health, Modeling of Noncommunicable Diseases Research Center, Hamadan University of Medical Sciences, Hamadan, Iran; 5 Department of Public Health, School of Public Health and Social Determinants of Health Research Center, Hamadan University of Medical Sciences, Hamadan, Iran

**Keywords:** Safe behaviors, Students, Health education, Theory of planned behavior

## Abstract

**Background:**

Behavior change interventions in tackling road traffic injuries are a public health concern. Thus, this interventional research was to survey the effect of safe traffic behaviors among male students in Hamadan, Iran, utilizing theory of planned behavior.

**Methods:**

In this quasi-experimental study, 204 students were randomly selected through multistage sampling from male high school students of Hamadan city*, west-central of Iran*, and non-randomly allocated to control and intervention groups (102 students in every group). The *self-administrate questionnaire w*as used for data collection in this research. Frequency (percentage) and mean (SD) were used for description. Cronbach alpha coefficient, content validity ratio (CVR) and content validity index (CVI) were used for psychometric evaluation of questionnaire and paired/independent sample t-test was used for data analysis. All statistical analyses were done in SPSS 19 and significant level was considered 0.05.

**Results:**

In both groups, more than 50 % of students walked to school. The two study groups were homogeneous in terms of confounding variables (*p* >  0.05). The validity of the questionnaire was confirmed and the total Cronbach’s alpha value was equal to 0.97. There was no significant difference in the score of safe traffic behaviors between the two groups before the intervention (*p* >  0.05). But after the intervention, the score in the intervention group was significantly increased (*p* < 0.05). Intragroup comparison also showed that only in the intervention group the score was significantly changed (*p* < 0.05).

**Conclusion:**

Theory of planned behavior is a suitable conceptual framework for planning the interventions to increase safe traffic behaviors in students.

## Background

Around 1.35 million people die in road accidents, more than 90% of whom die in road accidents live in low- and middle-income countries every year. Hence these countries are considered for more than 80% of the world’s population and only 48% of the world’s cars [1, 2]. In developing countries, traffic accidents place a heavy burden on the economy and cost about 3% of gross domestic product (GDP) for these countries [3] which equals to 2–4% of Iran’s GDP [4, 5].

In Iran, the third leading reason of death after coronary heart disease and stroke is the road traffic injuries (RTI) [6]. RTI is considered for 14.9% of all deaths and 26.9% of standard expected years of life lost (SEYLL) in Iran; hence, the death rate from these injuries was 58 per 100,000, especially in men [7]. A considerable section (35–50%) of RTI cost in Iran is associated to loss of productivity in terms of the premature death of victims at a young age as well as permanent or long-term disability of survivors due to spinal cord injury [8].

In fact, RTI as the first cause of death for under 40 years old people [9], especially people aged 10–24 years old [10] are adolescents or those who have never owned a car [11]. Besides, previous research indicated that young and male drivers in particular, regard themselves superior to others and are weak to assess their abilities and, consequently, in the precise evaluation of the risk of an accident [12]. As the similar way, Soori [13] reported boys’ are more vulnerable to traffic accidents than girls. Moreover, the range of death in the drivers under the age of 25 much more than older drivers in industrialized countries due to dangerous driving [14].

The share of the human element in the road accidents is reported to be from 50 to 80% which indicates the high significance of this component in road accidents [9]. Based on the statistics and the extent of factors influencing the accidents in Iran in 2015, 52% of people, 30% of road issues, and 13% of technical defects in cars were included in traffic accidents. Based on the report of Traffic Police Department, in 2015, about 156 thousand accidents were registered on the country’s roads, of which 54 thousand accidents, which is about one-third of the total number of accidents, were related to the reasons of road issues and about 81 thousand which considers for nearly half of all road accidents in 2015, was caused by human error [15].

Thus, the major focus must be on human elements and developing approaches to decrease human error. In reality, any intervention to increase safe traffic behaviors (STBs) must be handled to enhance knowledge, positive attitudes towards behavior, skills and understanding of traffic laws [16]. Therefore, research indicated that traffic education, especially for adolescents in most countries of world, has yielded valuable results [17].

By regarding people’s behavior significance in RTI occurrence, the STBs depends on various factors including perceptions, behaviors and attitudes of people. Theory of planned behavior (TPB) as a conceptual framework is one of the theories which can forecast occurrence of STBs [18]. Based on the TPB, people’s behaviors depend on constructs of subjective norms (SN), attitudes towards behavior (AB) and perceived behavioral control (PBC). The interventions can make next change in the intention and behavior by altering mentioned three constructs. Surveying 185 studies indicated that TPB model describes 39% of intention variance and 27% of behavior variance [19]. Considering road safety behaviors, knowledge and awareness of an individual are known as protective factors in the prevention of RTI according to the good performance of TPB theory in the field of traffic accidents, which has been confirmed in many studies [12, 20–22]. Therefore, our study seeks to answer the question of whether TPB-based educational interventions can improve STBs in students. If this educational intervention is effective, it can be provided as a necessary education in high schools to reduce potential traffic hazards.

Since Iran has a high rate of RTI and men and young people are more likely to suffer from RTI, so this study aimed to envestigate educational intervention effect on STBs by using TPB in male students of Hamadan, *west-central of Iran.*

## Methods

### Study design

**Fig. 1:**
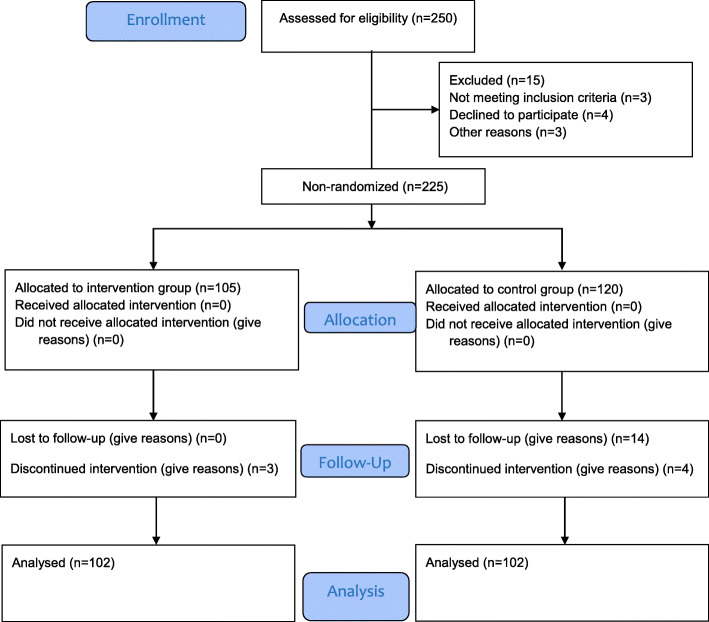
The CONSORT flow diagram of study

In this quasi- experimental study as shown in Fig. 1, 204 students were randomly selected through multistage sampling from Hamadan city (*west-central of Iran*) high schools and non-randomly allocated to control and intervention groups (102 students in every group). After coordination with the department of education and law enforcement agencies, students in the academic year of 2020–2021 from October to January 2020 were entered in the research with the informed consent of all participants and/or their legal guardians (if participants were under 16 years age). At the starting of research, the parental consent form was completed. The students participating were informed about the confidentiality of information.

### Instrument

A questionnaire was applied as the tool in the present research and the research team designed it online, and its validity and reliability was monitored. The questionnaire contained 10 questions related to the demographic features of students, the knowledge questions involved 5 four-choice questions with minimum and maximum scores of 0 and 5, respectively (e.g. When can a pedestrian cross the pedestrian crossing? Answers a) When in a hurry, he can pass regardless of the traffic light b) When the pedestrian traffic light is green c) When the pedestrian traffic light is red d) None, which correct and incorrect answers were scored 1 and 0, respectively.

The questions which were designed based on TPB with a five-point Likert scale are presented in Table 1. In addition to the 24 questions presented in Table 1, 6 questions about traffic behavior knowledge were included in the questionnaire.

**Table 1:** Question items based on theory of planned behavior with a five-point Likert scale

TPB constructs	Questions	1	2	3	4	5
Attitude^a^	1. Fastening the seat belt in the car will prevent my injury.					
	2. It is pleasant for me to wear a seat belt in the car.					
	3. Using a helmet while cycling will prevent my injury.					
	4. It is pleasant for me to use a helmet while cycling.					
	5. Crossing the sidewalk, escalator, and underpasses allowed to go to school will prevent my injury.					
	6. It is pleasant for me to cross the pedestrian crossings, escalators and authorized underground passages.					
Subjective Norms^a^	1. People who are important to me recommend that I fasten my seat belt in the car.					
	2. People who are important to me support me to fasten my seat belt in the car.					
	3. People who are important to me recommend that I wear a helmet while cycling.					
	4. People who are important to me support me to wear a helmet when cycling.					
	5. People who are important to me recommend that I go through pedestrian crossings, escalators, and underpasses.					
	6. People who are important to me support me in crossing pedestrian crossings, escalators and authorized underground passages.					
Perceived behavioral control^a^	1. It is easy for me to use a seat belt in the car.					
	2. I can fasten my seat belt in the car in any situation.					
	3. It is easy for me to use a helmet while cycling.					
	4. I can use a helmet in any situation while cycling.					
	5. It is easy for me to cross pedestrian crossings, escalators and authorized underground passages.					
	6. I can cross pedestrian crossings, escalators and authorized underground passages in any situation.					
Behavioral intention^a^	1. I intend to do safety traffic behavior more.					
	2. I intend to fasten my seat belt in the car.					
	3. I intend to use a helmet when cycling.					
Behavior^b^	1. I fasten my seat belt in the car.					
	2. I wear a helmet when cycling.					
	3. I cross pedestrian crossings, escalators and authorized underground passages.					

^a^5- Strongly Agree, 4- Somewhat Agree, 3- Neither Agree Nor Disagree, 2- Somewhat Disagree, 1-Strongly Disagree

^b^5- Always, 4- Often, 3- Sometimes, 2- Rarely, 1- Never

### Reliability and validity of instrument

The face validity of the questionnaire was checked and confirmed by 10 students. Content validity index and content validity ratio both were higher than 0.79, so content validity of instrument approved. Cronbach alpha coefficient was acceptable (0.95). The Cronbach alpha coefficient for knowledge, subjective norms, attitude towards behavior, behavioral intention, perceived behavioral control constructs were 0.98, 0.92, 0.98, 0.95, and 0.87, respectively. The test-retest reliability of the questionnaire was confirmed (Pearson correlation coefficient = 0.85 and interaclass correlation coefficients = 0.83).

### Sample size and sampling procedure

To determine the sample size, according to a study by Hemmati et al. (2017), Which examined safe behaviors in middle school students in Qom, Iran, only 46% of boys have safe behavior in road crossing so in following formula we can consider p equal to 0.46. With considering the significance level of 5% (α = 0.05), and the error rate of 0.13, the initial sample size in each group was equal to 54. According to the multi-stage sampling design, after multiplying the initial sample size by a design effect of 1.5 and 20% non-response rate, the total sample size was considered 102 people in each group.

$$ {\displaystyle \begin{array}{l}\kern0.72em p=0.46,\alpha =0.05,d=0.13,\kern0.36em {n}_0\ge \frac{z_{1-\frac{\alpha }{2}}^2\;p\left(1-p\right)}{d^2}=54\\ {}n={n}_0\times design\kern0.17em effect=54\times 1.5=81\left.\begin{array}{l}\\ {} non\kern0.17em response\kern0.17em rate=20\%\end{array}\right\}{n}_{Final}=\frac{n}{1- non\kern0.17em response rate}=\frac{81}{1-0.2}=101.25\end{array}} $$

The sampling method was multi-stage. In the first step, by cluster random sampling method, two districts were randomly selected from the four districts of Hamadan city, and then the list of boys’ high schools in each district was extracted. In the second step, two schools (one public school and one private school) from each region were randomly selected by cluster random sampling. In the third step, to get the sample share to be taken from each school the total number of students in grades 7 to 12 in each school was calculated and divided into the total number of students in grades 7 to 12 in four schools. The calculated number was multiplied by the sample size that was initially calculated. In the fourth step, the required number of samples for sampling from each school was determined. Inside each school, sampling was done by stratified random sampling with proportion to the size of students in each grade. The participants were non-randomly assigned to the study groups based on each individual’s interest.

### Conceptual framework

**Fig. 2:**
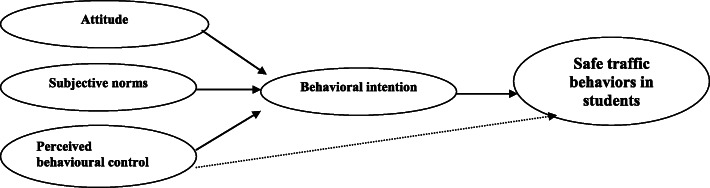
Conceptual framework of the theory of planned behavior in high school students [18]

The TPB conseptual framework in our study is presented in Fig. 2.

### Inclusion and exclusion criteria

In this study, the inclusion criteria of students were seemingly recognizable physical and mental health, no history of accidents, with the informed consent from all participants and/or their legal guardians (if participants were under 16 years age). Exclusion criteria was unwillingness to cooperate during the study or parental dissatisfaction.

### Intervention procedure

The educational content in terms of the requirement evaluation in the pre-test of current research and scientific and valid sources was identified. The educational content involved (1) an educational video (researcher lecture with using proper photos and animations) (2) a 20-page educational booklet entitled “Educational booklet to enhance the level of student traffic safety” and also (3) an educational pamphlet to enhance student STBs. The educational intervention was performed as follows in six steps.

- Step 1: The research team designed the educational contents by applying experts’ opinions with an emphasis on effective constructs of TPB and using appropriate strategies to change them.
- Step 2: Before the intervention, online questionnaires of STBs were distributed among the two groups and completed by them.
- Step 3: Videos, books and pamphlets related to STBs were distributed to the intervention group. No action was taken for the control group at this stage.

The intervention group, consisting of 102 students was divided into 6 groups based on the educational level for the educational intervention to be more effective. The 3 educational sessions (due to using the educational booklet and video) handled for the intervention group were approximately one hour in the form of group sessions of about 17 people (18 sessions in total). The researcher, within a month, provided one lecture session for the parents of these students (as one of the most important subjective norm references) in the form of group sessions in the network social media (6 sessions in total). Throughout of the teaching approaches, lectures, presenting the educational booklets, distribution of educational videos, group discussions, questions and answers were used.

- Step 4: One month after the educational intervention to follow and review the educational program, one educational reminder session was also handled applying the social networks in 6 groups of about 17 students for the intervention group (6 sessions in total). Hence, the total educational sessions received to 30 sessions.
- Step 5: Two months after the educational reminder session and review, the post-test was handled in both groups by applying the questionnaire in social media.
- Step 6: After gathering the post-test questionnaires, a one-hour session involving educational video, question and answer, and group discussion applying social networks was handled in the control group (6 sessions in total).

### Statistical analysis

Frequency and percentage, mean and standard deviation were used to describe the variables. Before performing inferential statistics, first the normality of quantitative variables was checked by Kolmogorov-Smirnov test and after confirmation, two independent samples, paired t-test and chi-square and fisher exact test were used. All analyzes were performed in SPSS 19 software with a significance level of 5%.

## Results

The highest frequency due to the educational level in studying groups was related to the seven educational levels with 20.6% in the intervention group and the eighth and ninth educational levels with 17.6% in the control group (Table 2). Considering the results in the intervention group, 55.9% of samples were educated in public schools and 44.1% in non-governmental schools, and in the control group, 52% were in public schools and 48% in non-governmental schools. The highest frequency in relation to the number of students’ family members in the study groups was less than or equal to three, with 31.4% in the intervention group and 5 with 32.4% in the control group. The highest frequency of father occupations in the study participants in intervention with 43.1% and control with 36.3% groups related to self-employed and also the highest frequency of mother occupations in the intervention and control groups related to housekeeping with 37.3 and 38.2%, respectively. The highest frequency of education of the father of participants in the intervention (34.3%) and control (34.3%) groups was a bachelor. The highest frequency of the mother education of the participants in the intervention group (33.3%) was diploma and bachelor and in the control group (35.3%) was a bachelor. Moreover, the highest frequency of school commuting among study participants in the intervention (51.0%) and control (54.9%) groups was related to pedestrians.

**Table 2:** Frequency and percentage of demographic variables

Characteristics		Intervention		Control	
		N	%	N	%
Grade	7	21	20.6	17	16.7
	8	17	16.7	18	17.6
	9	19	18.6	18	17.6
	10	15	14.7	16	15.7
	11	18	17.6	17	16.7
	12	12	11.8	16	15.7
Type of previous school	Public	57	55.9	53	52
	Private	45	44.1	49	48
Number of family members	3≥	32	31.4	28	27.5
	4	19	18.6	21	20.6
	5	30	29.4	33	32.4
	6≤	31	20.6	20	19.6
Father’s job	Self-employed	44	43.1	37	36.3
	Employee	22	21.6	19	18.6
	Manual worker	16	15.7	20	19.6
	Driver	10	9.8	14	13.7
	Retired	8	7.8	8	7.8
	Other	2	2	4	3.9
Mother’s job	Self-employed	14	13.7	9	8.8
	Employee	37	26.3	31	30.4
	Manual worker	4	3.9	10	9.8
	Driver	4	3.9	3	2.9
	Retired	5	4.9	10	9.8
	Housewife	38	37.3	39	38.2
Father’s education level	Below diploma	18	17.6	11	10.8
	Diploma	29	28.4	21	20.6
	Associate Degree	15	14.7	26	25.5
	Bachelor	35	34.3	35	34.3
	Master	5	4.9	9	8.8
Mother’s education level	Below diploma	12	11.8	10	9.8
	Diploma	34	33.3	24	23.5
	Associate Degree	18	17.6	22	21.6
	Bachelor	34	33.3	36	35.3
	Master	4	3.9	10	9.8
Transportation mode from home to school	On foot	52	51	56	54.9
	Bike	24	23.5	22	21.6
	Public transportation	17	16.7	13	12.7
	A combination of three methods	9	8.8	11	10.8
Age	Mean (SD)	17.31 (2.14)		16.83 (3.05)	

The study of homogeneity of study variables in the two groups showed that the distribution of variables in the two study groups did not differ significantly (*P* >  0.05). In the other word, according to the results of Chi-square test, the two groups did not differ significantly in terms of the variance of education level. The comparison of the effectiveness of the intervention is presented in Table 3 by comparing the scores before and after the intervention.

**Table 3:** Comparison the distribution of the TPB constructs between intervention and control groups

Constructs of theory	Group	Before the intervention	After the intervention	*P-value
		Mean (SD)	Mean (SD)	
Knowledge	Intervention	2.39 (0.91)	4.49 (0.59)	> 0.001
	Control	2.47 (0.97)	2.53 (1.01)	0.395
	^**^P-Value	0.553	> 0.001	
Attitude toward behavior	Intervention	15.23 (3.17)	24.32 (3.32)	> 0.001
	Control	15.51 (4.20)	14.89 (3.38)	0.063
	^**^P-Value	0.586	> 0.001	
Subjective norm	Intervention	11.79 (4.02)	23.20 (3.73)	> 0.001
	Control	12.61 (4.83)	12.68 (4.60)	0.746
	^**^P-Value	0.187	> 0.001	
Perceived behavior control	Intervention	15.21 (2.73)	24.69 (2.68)	> 0.001
	Control	15.61 (3.92)	15.82 (3.64)	0.453
	^**^P-Value	0.397	> 0.001	
Behavioral intention	Intervention	7.73 (1.91)	12.67 (1.76)	> 0.001
	Control	7.93 (2.24)	8.05 (2.12)	0.411
	^**^P-Value	0.503	> 0.001	
Behavior	Intervention	8 (1.59)	13.07 (1.50)	> 0.001
	Control	8.17 (2.32)	8.08 (2.20)	0.563
	^**^P-Value	0.528	> 0.001	

*The results are related to paired sample t-test

** The results are related to independent sample t-test

According to Table 3, results of two independent sample t-test in comparing two groups before intervention showed the mean scores of knowledge (*p* = 0.553), attitude towards the behavior (*p* = 0.586), subjective norms (*p* = 0.187), perceived behavioral control (*p* = 0.397), behavioral intention (*p* = 0.503) and behavior (*p* = 0.528) between the control and intervention groups was not statistically significant before the intervention.

Results of paired t- test in control group showed the mean scores of knowledge (*p* = 0.395), attitude towards the behavior (*p* = 0.063), subjective norms (*p* = 0.746), perceived behavioral control (*p* = 0.453), behavioral intention (*p* = 0.411) and behavior (*p* = 0.563) in the control group was not statistically significant before and after the intervention (Table 2).

Results of paired t- test in intervention group showed the mean scores of knowledge (*p* < 0.001), attitude towards the behavior (*p* < 0.001), subjective norms (*p* < 0.001), perceived behavioral control (*p* < 0.001), behavioral intention (*p* < 0.001) and behavior (*p* < 0.001) in the intervention group was statistically significant before and after the intervention (Table 2).

Results of two independent sample t-test in comparing two groups after intervention showed the mean scores of knowledge (*p* < 0.001), attitude towards the behavior (*p* < 0.001), subjective norms (*p* < 0.001), perceived behavioral control (*p* < 0.001), behavioral intention (*p* < 0.001) and behavior (*p* < 0.001) between the control and intervention groups was statistically significant after the intervention (Table 3).

## Discussion

This research was performed to survey the impact of educational intervention on the basis of TPB on STBs of Hamadanian students in Iran. The results indicated that constructs of the TPB considerably increased after the educational intervention in the intervention group, but this increase was not considerable for the control group. Also, statistically significant differences were observed between intervention and control groups after the intervention in the constructs.

TPB constructs can be used to predict human behaviors which has been widely used in different studies. For example, Li et al. used the theory of planned behavior to study the high-risk behaviors of truck drivers in 2021. Their results showed that among perceived behavioral control, subjective norm and attitude toward high-risk behavior, attitude toward high-risk behavior is the strongest predictor in creating high-risk behaviors in truck drivers [23]. Consistenly, Piazza et al. used TPB and found that attitude toward behavior was the most important factor of intention to use mobile device while crossing the street [24]. Inconsistly, Jiang et al. have found that influence of subjective norm and attitude is weak in comparison to distraction perception and behavioral intention to use mobile devise while cycling [25]. Man et al. have revealed that construct of perceived behavioral control is correlated with the risk-taking behavior of workers [26]. Ledesma et al. in 2018 used TPB to predict seatbelt use behaviors, which they showed that the components of TPB play an important role in predicting seatbelt use behaviors [27]. Poulter and McKenna in their research on students aged 15 to 16 years, indicated the effect of an educational intervention on the basis of TPB on beliefs related to STBs [28].

In Iran, [29] indicated that the school-based educational intervention was efficient on the safe crossing of streets in students. Hemmati and Gharlipour in 2017 reported that application of TPB can increase STBs in road crossing [20]. Ramezankhani et al. 2014 indicated that the design and implementation of educational programs on the basis of TPB could significantly improve street-crossing behaviors such as crossing one-way, two-way streets, crossing pedestrian bridges, crossing pedestrian lights, not rushing when crossing (at the rate of 15%) [17]. Nazari 2008 reported 13% of cross-street behaviors in the elementary students after the educational intervention on the basis of the precede-proceed model and TPB [21].

The intervention handled in this research involved researcher lecture by using proper photos and animations, educational booklet and pamphlet to enhance the level of students’ traffic safety, group discussions, questions and answers. These interventions increased students’ awareness of STBs. At the same time, their attitudes toward these behaviors improved. Moreover, they received negative reviews for high-risk traffic behaviors. Behavioral intention and behavior also increased as self-efficacy and behavioral control among peers about STBs. Therefore, it was indicated that training and obtaining information in a specific field to people who are significant to the person is a wave that increases the likelihood of confirmation of the behavior by them [30].

Thus, teaching traffic behaviors, if performed in a combination of theoretical and practical methods, can have a positive influence on enhancing the STBs because it engages various senses in training, and the more senses are included in training, the more learning and retention is learned. On the other hand, education in the form of games and entertainment can be efficient to attract children’s attention and promoting their learning. It should not be overlooked that education is a continuous and dynamic process through which members of society, especially adolescents, can learn the roles, expectations, rules, and relationships and in general the culture of society for survival, the theoretical-practical educational method at the school level can be a long and efficient step to decrease traffic hazards in young students.

For gathering data a self-report tool was applied that could be related to the participants’ bias for obtaining the answers related to social desirability factor. Hence, there will be more reliable results by direct observation of STBs. Besides, this study’s results were constricted to Hamadanian students which show cautions to generalize the results to other age and gender groups.

## Conclusions

TPB is a beneficial theory to plan the interventions for increasing the STBs. Families, schools, and other relevant institutions can bypass protocols to enhance the STBs by affecting the structures of TPB to enhance the STBs like using helmets when applying bicycles, not rushing when crossing the street, not utilizing cell phones while biking or crossing the street and apply pedestrian bridge in the students.

## Data Availability

The datasets used and analyzed during the current study are available from the corresponding author on reasonable request.

## Abbreviations

TPB: theory of planned behavior
STB: safety traffic behavior
CVR: content validity ratio
CVI: content validity index
RTI: road traffic injuries
GDP: gross domestic product
SEYLL: standard expected years of life lost

## References

[1] World Health Organization, Global Status Report on Road Safety 2018. Geneva: Summary, World Health Organization; 2018.

[2] Mostafavi F, Nasirian M, Zeinali M, Ardalan G, Mohebpour F, Daniali SS, Pirzadeh A, Kelishadi R (2021). Evaluating community-based programs in promoting traffic behaviors and safe road crossing behaviors in youth: an application on theory of planned behavior. Int J Prev Med.

[3] Nguyen-Phuoc DQ, De Gruyter C, Nguyen HA, Nguyen T, Su DN: Risky behaviours associated with traffic crashes among app-based motorcycle taxi drivers in Vietnam. Transport Res F: Traffic Psychol Behav 2020;70:249–59. 10.1016/j.trf.2020.03.010.

[4] Hasanzadeh J, Moradinazar M, Najafi F, Ahmadi-Jouybary T (2014). Trends of mortality of road traffic accidents in Fars Province, southern Iran, 2004-2010. Iran J Public Health.

[5] Rezaei S, Arab M, Matin BK, Sari AA (2014). Extent, consequences and economic burden of road traffic crashes in Iran. J Injury Violence Res.

[6] Meskarpour Amiri M, Bahadori M, Mehrabi-Tavana A (2017). The dilemma of road traffic accidents in Iran. Int J Med Rev.

[7] Akbari M, Naghavi M, Soori H (2006). Epidemiology of deaths from injuries in the Islamic Republic of Iran. EMHJ-Eastern Mediterranean Health J.

[8] Vahdati SS, GhafarZad A, Rahmani F, Panahi F, Rad AO (2014). Patterns of road traffic accidents in north west of Iran during 2013 new year holidays: complications and casualties. Bull Emerg Trauma.

[9] Javadi S-M-H, Tahmasebi S, Azari-Arghun T, Arshi M, Alipour F (2017). The youth and experience of traffic accidents (grounded theory). Q J HealthAcc Disasters.

[10] Toroyan T, Peden M (2007). Youth and road safety.

[11] Ahmed SS, Pantangi SS, Eker U, Fountas G, Still SE, Anastasopoulos PC (2020). Analysis of safety benefits and security concerns from the use of autonomous vehicles: a grouped random parameters bivariate probit approach with heterogeneity in means. Anal Method Acc Res.

[12] Dıaz EM: Theory of planned behavior and pedestrians' intentions to violate traffic regulations. Transport Res F: Traffic Psychol Behav. 2002;5(3):169–75, DOI: 10.1016/S1369-8478(02)00015-3.

[13] Soori H (2002). Epidemiology of children's cycling injuries in Ahwaz, Islamic Republic of Iran. EMHJ-Eastern Mediterranean Health J.

[14] Thelin R. Indiana Traffic Safety Facts: Dangerous Driving 2019.*.* 2020.

[15] Agency Mn. Death in the ambush of Iranian drivers / ranked 189th among 190 countries. 2016. Available at: https://www.mehrnews.com.

[16] Riaz MS, Cuenen A, Dhondt S, Craps H, Janssens D, Wets G, Brijs T, Brijs K (2019). Evaluation of a road safety education program based on driving under influence and traffic risks for higher secondary school students in Belgium. Safety.

[17] Ramezankhani A, Nilsaz M, Dehdari T, Soori H, Tavasoli E, Khezli M, Zinat Motlagh F (2014). Effects of an educational intervention based on planned behavior theory in promoting safe behaviors crossing the street in students. J Health System Res.

[18] Ajzen I (1991). The theory of planned behavior. Organ Behav Hum Decis Process.

[19] Armitage CJ, Conner M (2001). Efficacy of the theory of planned behaviour: a meta-analytic review. Br J Soc Psychol.

[20] Hemmati R, Gharlipour Z (2017). Study of the safe behavior in road crossing using the theory of planned behavior among middle school students. Int J Pediatr.

[21] Nazari M. The integration of precede-proceed model with theory of planned behavior for promoting safety behaviors among child pedestrians Tehran. School of Medical Sciences: Tarbiat Modares University. 2008:1.

[22] Dong X, Peek-Asa C, Yang J, Wang S, Chen X, Chi G, Ramirez M (2011). The association of road safety knowledge and risk behaviour with paediatric road traffic injury in Guangzhou, China. Inj Prev.

[23] Li Z, Man SS, Chan AHS, Zhu J (2021). Integration of theory of planned behavior, sensation seeking, and risk perception to explain the risky driving behavior of truck drivers. Sustainability.

[24] Piazza AJ, Knowlden AP, Hibberd E, Leeper J, Paschal AM, Usdan S (2019). Mobile device use while crossing the street: utilizing the theory of planned behavior. Accid Anal Prev.

[25] Jiang K, Yang Z, Feng Z, Yu Z, Bao S, Huang Z. Mobile phone use while cycling: a study based on the theory of planned behavior. Transport Res F: Traffic Psychol Behav. 2019;64:388–400. 10.1016/j.trf.2019.05.020.

[26] Man SS, Chan AHS, Alabdulkarim S (2019). Quantification of risk perception: development and validation of the construction worker risk perception (CoWoRP) scale. J Safety Res.

[27] Ledesma RD, Tosi JD, Díaz-Lázaro CM, Poó FM (2018). Predicting road safety behavior with implicit attitudes and the theory of planned behavior. J Safety Res.

[28] Poulter DR, McKenna FP (2010). Evaluating the effectiveness of a road safety education intervention for pre-drivers: an application of the theory of planned behaviour. Br J Educ Psychol.

[29] Tolide M, Dehghani Tafti A, Rahaei Z, Eisapareh K: The Impact of a School-Based Educational Intervention on Safe Road Crossing in Elementary School Students. Payesh (Health Monitor). 2020:0–0.

[30] Parker D (2002). Changing drivers' attitudes to speeding: using the theory of planned behaviour.

